# The Relationship Between Chronic Pruritus, Attention-Deficit/Hyperactivity Disorder, and Skin Picking—A Case Series and Narrative Review

**DOI:** 10.3390/jcm14051774

**Published:** 2025-03-06

**Authors:** Eva Loos, Suzan Sekar, Christiane Rosin, Alexander A. Navarini, Chrysovalandis Schwale, Rainer Schaefert, Simon Müller

**Affiliations:** 1 Department of Dermatology, University Hospital and University of Basel, CH-4031 Basel, Switzerlandsimon.mueller@usb.ch (S.M.); 2 Department of Psychosomatic Medicine, University Hospital and University of Basel, CH-4031 Basel, Switzerlandrainer.schaefert@usb.ch (R.S.)

**Keywords:** pruritus, ADHD, skin picking, itch, itching, psychodermatology, psychotherapy, mental, scratching, prurigo nodularis, psychometric assessment, screening tools

## Abstract

Chronic pruritus (CP), attention-deficit/hyperactivity disorder (ADHD), and skin picking disorder (SPD) are medical conditions that involve both somatic and psychosocial dimensions, posing unique challenges in clinical management. While CP and SPD are often observed together, the link between ADHD and these conditions is less recognized. This conceptual work describes three women who suffered from a complex interplay of CP, ADHD, and SPD treated at our specialized bi-disciplinary psychodermatological pruritus clinic. Based on our clinical observation and a narrative review of the literature, we assume a bidirectional, triangular relationship between CP, ADHD, and SPD. To support this assumption, we propose two hypotheses: (1) a neurodevelopmental hypothesis, emphasizing that an underlying neurodevelopmental disorder, in this case, ADHD, might present with symptoms like dysfunction of sensory processing, impulsivity, and attention deficits as shared features that reinforce CP and SPD, and (2) a neuroinflammatory hypothesis, suggesting that similar neuroinflammatory signatures promote the co-occurrence of CP, ADHD, and SPD. In addition, we provide specific suggestions derived from our clinical experience on how to manage patients with this complex combination of conditions. Elucidating the interplay between CP, ADHD, and SPD might help develop personalized treatment strategies and improve outcomes.

## 1. Introduction

Chronic pruritus (CP), attention-deficit/hyperactivity disorder (ADHD), and skin picking disorder (SPD) are medical conditions with a somatic and psychosocial dimension associated with a high burden of disease. More information on the definition, prevalence, etiology, psychosocial aspects, mental comorbidities, and management of these three conditions is provided in Table 1.

Although seemingly disparate, CP, ADHD, and SPD can coexist in our experience, thereby posing a particular treatment challenge. While the association of CP and SPD can often be observed in clinical routine and has been described previously [1,2], the link between ADHD and either of these conditions is less recognized, and rarely described in the medical literature.

In the following, we present three female patients who suffered from a combination of CP, ADHD, and SPD and who were treated in our bi-disciplinary psychodermatological pruritus clinic at the University Hospital of Basel, Switzerland. In these patients, all three conditions seemed to mutually influence each other.

With this report, we aim to do the following:

(1) Raise awareness of the intricate interaction between CP, ADHD, and SPD;
(2) Generate hypotheses about the pathophysiological concepts underlying the triangular relationship of these conditions;
(3) Emphasize the benefits of a bi-disciplinary consultation setting involving dermatologists and mental health specialists and give recommendations from the clinical experience on how to recognize and manage these types of patients.

**Table 1:** Characteristics of chronic pruritus (CP), attention-deficit/hyperactivity disorder (ADHD), and skin picking disorder (SPD); information in this table refers to adults.

	Chronic Pruritus (CP)	Attention-Deficit/Hyperactivity Disorder (ADHD)	Skin Picking Disorder (SPD)
**Definition**	Pruritus is an unpleasant sensation that provokes the desire to scratch [3]. CP is defined by a duration of ≥6 weeks [4].	ADHD is a neurodevelopmental disorder defined and characterized by a persistent pattern of inattention and/or impulsivity and hyperactivity [5].	SPD, also known as excoriation disorder/dermatillomania, is characterized by recurrent and compulsive picking of the skin, despite repeated attempts to stop it [5]. In the ICD-11, SPD falls under the category of bodily focused repetitive behaviors as part of the obsessive–compulsive spectrum.
**Prevalence**	Approx. 20% of the general population in Western countries during their lifetime [6,7].	Approx. 3% in adults [8].	Approx. 4% of the general population, with women being more affected than men [9].
**Etiology**	Caused by numerous dermatological and non-dermatological diseases (e.g., chronic kidney disease, hepatobiliary disorders, thyroid disease, iron deficiency, polyneuropathy, psychiatric conditions, malignancies, pruritogenic medication) [10].	Primarily recognized in childhood and often persisting into adulthood. Underlying factors are not completely understood yet, possibly a combination of genetic predisposition and pre-, peri-, and postnatal environmental factors impacting brain development [11].	Results from an urge to find relief from uncomfortable sensations (e.g., itch) or inner tension caused by distress or aversive emotions (e.g., shame, anger, helplessness, anxiety, boredom) [12,13,14].
**Psychosocial aspects**	High psychosocial burden due to distress and impaired quality of life. Excessive scratching and skin manipulation are often accompanied by a pleasurable urge to scratch [15,16], which drives the vicious itch–scratch cycle, thereby aggravating CP.	Impaired functioning and quality of life in many areas of life comprising education/profession or social interactions (partner, family, friends).	Skin damage, presenting as wounds, crusts, or nodes, can result in distress (e.g., through social stigmatization) and reinforce the picking behavior—even in the absence of cutaneous sensations, such as pruritus or pain.
**Mental comorbidities**	Insomnia, depression, anxiety disorders, obsessive–compulsive disorders, somatic symptom disorder, or ADHD [17,18,19,20].	Substance abuse and addiction, affective disorders, eating disorders. Borderline personality disorder is considered to be a possible differential diagnosis [11].	Trichotillomania, generalized anxiety disorder, depression, ADHD, obsessive–compulsive disorders [21].
**Management**	(1) General: use of emollients, avoidance of skin dryness, irritants, and stress (2) Pharmacological: topical and systemic drugs according to current guidelines [10,22] (3) Non-pharmacological, device-based interventions: e.g., phototherapy, and in selected cases, laser, neurostimulation, cryotherapy, acupuncture [23]	(1) Psychological: psychoeducation, psychotherapy (2) Pharmacological: stimulants (methylphenidate, amfetamine, lisdexamfetamine) (3) Neurofeedback [11]	(1) Topical treatment: anti-inflammatory (if the skin is inflamed), itch-relieving emollients, occlusion with bandages or band-aids (2) Cognitive behavioral therapy (CBT) (3) Pharmacological: selective serotonin reuptake inhibitors (e.g., fluoxetine, escitalopram), lamotrigine, glutamatergic agents (N-acetylcysteine) [24,25]

## 2. Case 1

### 2.1. Patient History

A 53-year-old former bank employee had been suffering from CP for six years without a discernible cause. Initially, she had scratched only when experiencing an itch, but soon after, she began picking and manipulating wound crusts with sharp tools like scissors, knives, or fingernails, even in the absence of an itch. She stated that this behavior stemmed from an irresistible urge to manipulate her skin. This manipulation had a calming effect, as it allowed her to focus on the act of picking, contrasting with her usual inattentive and disorganized nature. As a result of her CP and skin manipulation, she experienced a significantly reduced quality of life. She also reported considerable distress and anxiety related to ongoing family issues. Recently, two of her three children had been diagnosed with ADHD, and she suspected she might have ADHD as well. The patient had a history of panic disorder, for which she had been receiving escitalopram (10 mg/d), but never underwent psychotherapy previously. Prior treatment had consisted of topical corticosteroids, pimecrolimus, polidocanol-containing moisturizers, occasional oral bilastine (20 mg/day), and narrowband ultraviolet B (NB-UVB) phototherapy (three times a week for three months).

### 2.2. Dermatological and Psychological Assessments

At the first consultation in our joint bi-disciplinary pruritus clinic, quantitative and qualitative itch assessments were performed, along with screening for mental health-related issues, such as depression, anxiety, and somatic distress (refer to Table 2 for an overview of all assessment tools and corresponding scores for each patient). The patient reported a high average itch intensity over the past four weeks and described the itch quality as tickling, burning, and painful, indicative of a neuropathic component of her CP. The itch-related quality of life was reduced according to the German version of the ItchyQoL (GerItchyQoL), a questionnaire assessing the impact of CP on patients’ quality of life (e.g., daily activities, mood, and overall well-being) [26]. She presented with approx. 80 erythematous papules and nodules on her upper and lower extremities, as well as on her back, suggestive of chronic nodular prurigo (CNPG, also referred to as prurigo nodularis). This diagnosis was confirmed by lesional biopsy. The clinical presentation corresponded to an Investigator’s Global Assessment for Chronic Nodular Prurigo (IGA-CNPG) stage 3 (moderate). Of these lesions, more than 75% were excoriated or crusted, reflecting an activity stage 4 (severe) [26]. According to the Erlanger Score (also referred to as Diepgen-Score [27]), a diagnostic tool for evaluating the likelihood of atopic diathesis, there was no evidence of atopic predisposition. A pruritus laboratory screen was conducted according to the European guidelines on CP [10].Remarkable values for all cases are displayed in Table 2.

During the consultation, the patient appeared very stressed by her symptoms and psychosocial burden. She seemed restless, spoke rapidly and excessively, and frequently interrupted the clinicians’ explanations. The patient showed a clinically relevant score in the ADHD self-report questionnaire (ADHS-SB), which assesses ADHD-specific symptomatology (i.e., attention deficits, hyperactivity, and impulsivity) in adults according to DSM-IV, ICD-10 and 11. It is one of five subtests of the German Homburger ADHD Scale for Adults (HASE) [28]. The patient fulfilled the ICD-11 criteria for ADHD. The diagnosis was confirmed by an external psychiatrist specializing in ADHD. Additionally, SPD was diagnosed through further exploration and a high screening score in the modified Skin Picking Scale (mSPS-D), which evaluates the severity of skin picking with regard to, e.g., frequency, intensity, distress, and functional impairment [29]. The patient’s description of manipulation did not suggest any delusional content.

### 2.3. Applied Treatment and Further Procedures

Skin-directed treatment containing itch-relieving topicals was initiated (refer to Table 2). In addition, the patient participated in several individual in-house cognitive behavioral therapy (CBT) sessions focusing on itch-related aspects. As for ADHD, we started with methylphenidate (10 mg/d). Three weeks later, the patient reported a decreased itch intensity and improved impulse control. The latter may have led to a reduced urge to pick the skin, or it may have improved independently of any change to that urge. Although the patient continued to manipulate her skin in stressful situations, she became more aware of this maladaptive behavior and could process feelings more adaptively. The patient continued to have regular appointments in our clinic, as her symptoms were still not fully under control. In addition to her visits with us, she began external psychotherapy to work on her ADHD- and SPD-related difficulties. All these measures resulted in a significant and durable improvement with almost clear skin (IGA-CNPG severity and activity score ≤1).

## 3. Case 2

### 3.1. Patient History

A 69-year-old female artist presented in our clinic with CP persisting for over 10 years. Due to an uncontrollable, automatic urge to scratch, she had developed scarred, nodular, and dyspigmented skin lesions on her lower legs and arms. In addition to the significant itch-related strain, she experienced heightened mental distress due to the feeling of loneliness. The patient had a history of recurrent depressive disorder but never underwent psychopharmacological or psychotherapeutic treatment. She reported having difficulties organizing her appointments, losing track of time during activities, and frequently misplacing or searching for items. She mentioned that several professionals had previously suspected she might have ADHD, but no diagnostic evaluation had been conducted. Dermatological treatments had so far consisted of various topicals including creams containing clobetasol propionate, polidocanol, glycerin, and zinc. A previous lesional skin biopsy had shown spongiotic dermatitis.

### 3.2. Dermatological and Psychological Assessments

At the first visit to our clinic, the patient reported a moderate average itch intensity over the past four weeks and denied tingling, burning, or prickling itch sensations. Her itch-related quality of life was moderately impaired. She presented with several centimeter-sized, oval, partially excoriated nodules and plaques on her extremities, buttocks, and shoulders, suggestive of CNPG with stage 3 severity (moderate) and stage 4 activity (severe). Laboratory pruritus screening was conducted. During consultation, the patient behaved very restlessly, frequently interrupted conversations, and struggled to focus on medical explanations. She scored above the clinical cut-off on the ADHS-SB. ADHD diagnosis was confirmed by a specialized psychiatrist. She also met the ICD-11 criteria for SPD, with a clinically relevant mSPS-D score. During consultation, the patient reported no current depressive symptoms, which was consistent with the clinical presentation and the screening score (refer to Table 2).

### 3.3. Applied Treatments and Further Procedures

The patient was treated with itch-relieving topicals (refer to Table 2). Initially, she declined systemic medications including methylphenidate and was unable to adhere to the prescribed topical treatment, partly due to the disorganization associated with her ADHD and partly because of her personal rejection of conventional medicine. Eventually, she agreed to take *N*-acetylcysteine, which had been reported to have positive effects on SPD [35]. In addition, she received itch-focused in-house CBT. Subsequently, her skin picking subsided, and the skin lesions improved slightly, which the patient perceived as a significant relief. As her symptoms were still not under full control, she continued to have regular appointments at our clinic. The patient rejected starting outpatient psychotherapy. During the 18-month follow-up period, the IGA-CNPG fluctuated between stages 1 and 3, with an activity score ranging between 2 and 3.

## 4. Case 3

### 4.1. Patient History

A 51-year-old female unemployed laboratory assistant was referred to us with CP persisting for over two years, primarily affecting her arms and back. The intense itching caused her to scratch until her skin bled, but sometimes she would scratch also in the absence of itching. Furthermore, she reported the frequent urge to manipulate the skin due to “acne” since adolescence. She had been diagnosed with ADHD 20 years ago and had been taking methylphenidate (10 mg/d) ever since. Although this medication was somewhat effective, she continued struggling with ADHD-related symptoms, especially hypersensitivity to external auditory stimuli and impulsivity, the latter resulting in frequent interpersonal conflicts. Due to sleeping problems, she had been prescribed mirtazapine (15 mg/d), which had no positive effects. Previous dermatological treatments included creams containing mometasone 0.1% or polidocanol as well as a few sessions of NB-UVB light, which had not yielded significant improvement.

### 4.2. Dermatological and Psychological Assessment

The patient reported a high average itch intensity over the past four weeks and a severely reduced itch-related quality of life. The itch quality was described as tickling, painful, and biting. She presented no primary skin lesions, just a few scratch excoriations on her shoulders, lower trunk, and limbs, as well as multiple hypopigmented scars reflecting older, scarred scratch lesions. The laboratory pruritus screen yielded unremarkable results. The skin condition was ultimately determined as CP on primarily non-diseased skin (IFSI II), possibly facilitated by an atopic diathesis (Erlanger Atopy Score: 11/35).

Since the ADHD diagnosis had already been confirmed, no further specific screening was applied. The patient met ICD-11 criteria for SPD, with a clinically relevant value in the mSPS-D.

### 4.3. Applied Treatments and Further Procedures

Skin-directed treatment consisted of itch-relieving topicals and systemic treatment (refer to Table 2) and NB-UVB phototherapy, which was discontinued after a few sessions as symptoms worsened. Mirtazapine was stopped, due to its lack of benefit on her sleep disturbances, but the patient refused alternative antidepressants. The pre-existing medication with methylphenidate remained unchanged. She was referred to an external psychotherapist specializing in ADHD, where she learned helpful strategies and techniques to reduce ADHD-related symptoms and associated negative psychosocial consequences. During an 11-month follow-up, the itch intensity fluctuated but never reached peaks as high as in the beginning (average 6/10). The patient reported manipulating her skin less often.

## 5. Discussion

We herein presented three women experiencing a combination of chronic pruritus (CP), attention-deficit/hyperactivity disorder (ADHD), and skin picking disorder (SPD)—conditions that seem to influence each other in a bidirectional manner. Although we see more cases with this combination of conditions in our clinical practice, we chose to focus on the three most salient examples. Nonetheless, a larger patient sample is required to further substantiate the two hypotheses proposed in the following discussion.

**Figure 1:**
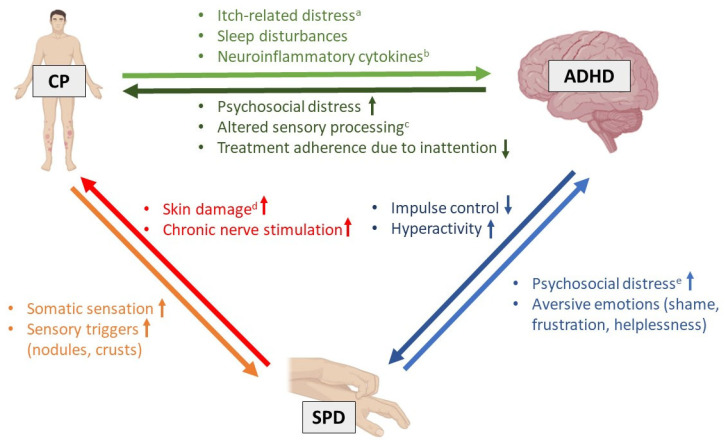
Suggested bidirectional, triangular relationship between chronic pruritus (CP), attention-deficit/hyperactivity disorder (ADHD), and skin picking disorder (SPD) as observed in our three female cases. ^a^ e.g., fatigue, anxiety, irritability, avoidance, worrying, social withdrawal; ^b^ e.g., interleukin (IL)-6, IL-4, IL-17A, nerve growth factor; ^c^ e.g., hypo-/hyper-responsiveness of touch, clothing, external irritants (cosmetics, disinfectants, detergents); ^d^ e.g., inflammation, crusts as observed in prurigo nodularis or lichen simplex chronicus; ^e^ e.g., interpersonal conflicts (e.g., family, job), stigmatization, avoidance behavior.

The CP-related SPD observed in our patients might have been facilitated by ADHD-associated impaired impulse control. Likewise, attention deficits, as part of the patients’ ADHD, may have led to difficulties adhering to dermatological treatments, thereby worsening CP and SPD. This in turn might have exacerbated ADHD symptoms through itch-related distress and sleep disturbances. That vicious cycle of possible interactions is illustrated in Figure 1.

To our knowledge, the triangular relationship of CP, ADHD, and SPD has not yet been described in the literature, but a few studies indicated that certain bidirectional interactions do exist:

- *Bidirectional relationship between CP and ADHD*: Studies have shown that children with atopic dermatitis (AD), a chronic inflammatory skin disease in which CP is a cardinal symptom, are more likely to have comorbid ADHD [36,37,38]. (Neuro)inflammatory processes may play a central role in this interaction. Proinflammatory serum cytokines (proteins serving as signaling molecules within the immune system) released in AD, such as interleukin (IL-)4, IL-6, IL-13, or TNF-α, can cross the blood–brain barrier and potentially interfere with the maturation of prefrontal cortex regions and neurotransmitter systems involved in the pathophysiology of ADHD [39,40,41,42,43]. However, there is limited research on the association between ADHD and other itchy dermatoses, such as psoriasis, urticaria, and prurigo [36]. This may be because AD and ADHD typically manifest during childhood, whereas other forms of CP often emerge later in life when ADHD may be less apparent.
- *Bidirectional relationship between ADHD and SPD*: It was hypothesized that the repetitive, compulsive nature of SPD shares similarities with the impulsivity seen in ADHD [44]. ADHD has been previously reported in individuals with SPD, with prevalence rates of ADHD in 8–12% of SPD patients [21,45]. These publications suggest potential shared neurobiological mechanisms between these two disorders; however, they do not elaborate further on potential mechanisms. In addition, these studies were cross-sectional, thereby limiting statements of the causality of temporal relationships. Longitudinal studies are needed to understand the interaction of these disorders over time.
- *Bidirectional relationship between CP and SPD*: According to a single-center, cross-sectional study from China, SPD was associated with higher itch intensities and was prevalent in about 65% of patients with AD and about 29% of patients with psoriasis [2]. The authors surmised that the higher rate of SPD in AD reflects the higher prevalence and intensity of CP in these patients. Also, atopic skin might be more prone to react hypersensitively to environmental factors due to an impaired barrier and (latent) inflammation [46]. In turn, the severe itch can be aggravated through exacerbated skin damage caused by SPD, resulting in a perpetuated itch–scratch cycle in which scratch-induced inflammation causes more itching [47].

Combining our clinical observations with these findings from the literature, we propose two hypotheses, (a.) a neurodevelopmental hypothesis and (b.) a neuroinflammatory one, which aim to unify all three conditions through possible common factors.

### 5.1. Neurodevelopmental Hypothesis

This approach may be grounded in the close connection between skin and psyche, which has been assumed to originate from the shared development of the skin and nervous system from the same embryonic germ layer, the ectoderm [41]. Alterations in neuronal development, as seen in neurodevelopmental disorders like ADHD or autism spectrum disorder, can affect both the central and autonomic nervous systems, as well as the skin. For example, pathologic local alterations of the brain-derived neurotrophic factor levels might have a negative impact both on the development of the prefrontal cortex, which is crucial for executive functions (i.e., cognitive processes that regulate decision-making, behavior, activity level, impulse control, response inhibition, and attention) [48,49], and the morphology of neurons innervating the skin [50]. The combination of neurodevelopmental alterations can lead to (1) sensory processing problems, (2) hyperactivity and impulsivity, and (3) attention deficits, negatively affecting CP and SPD.

#### 5.1.1. Sensory Processing Problems

Sensory processing refers to how the brain actively predicts, organizes, and interprets sensory information from the body. This process allows individuals to generate adaptive responses to sensory information. Individuals with neurodevelopmental alterations typically exhibit dysfunctions in sensory and emotional processing [51,52,53], including altered sensitivity to touch [54]. This may make them more prone to experiencing itch and noticing skin irregularities, such as wounds or crusts, which can result in further scratching and manipulation [13,55]. In patients with ADHD, an unfiltered “sensory overload” may enhance itch sensation and trigger excessive scratching, hence worsening CP [56]. Conversely, the mental distress and sleep disturbances caused by heightened itch can exacerbate symptoms of ADHD [38,57].

#### 5.1.2. Hyperactivity and Impulsivity

In addition to heightened sensory sensitivity, hyperactivity and impulsivity are hallmarks of ADHD. The combination of increased itch sensation and poor impulse control can drive the itch–scratch cycle and, in the long run, lead to SPD. In patients with ADHD, skin manipulation through picking and scratching may serve as a coping mechanism for the distress caused by itch or by aversive emotions [12,13]. The temporary relief through skin picking further reinforces the vicious cycle that exacerbates CP, possibly resulting in secondary skin conditions such as prurigo nodularis or lichen simplex chronicus (IFSI group III), which are characterized by chronic scratch lesions. These lesions can again serve as sensory (and visual) triggers for pathological picking.

#### 5.1.3. Attention Deficits and Other Executive Dysfunctions

Aside from the mentioned impulsivity, deficits in executive functions in ADHD, like attention or response inhibition, can contribute to problems with treatment adherence. Moreover, mindless scratching and picking, especially when these behaviors become habitual [56], can make it even more difficult for patients to notice the skin picking and resist it.

**Figure 2:**
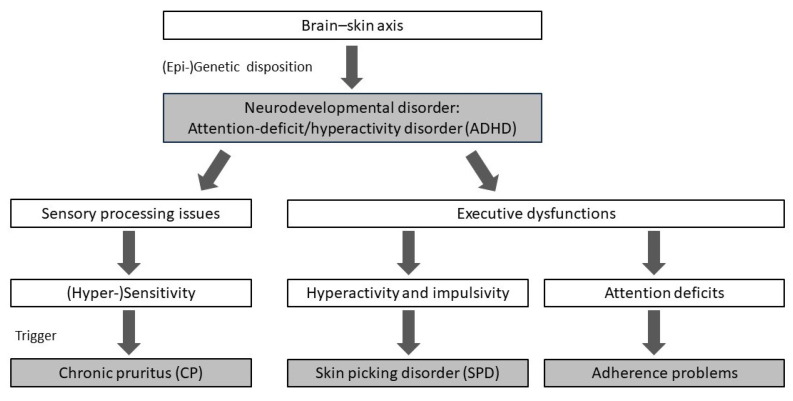
Proposed neurodevelopmental hypothesis. Attention-deficit/hyperactivity disorder (ADHD) as a neurodevelopmental disorder may result in difficulties with sensory processing and executive functions, like hyperactivity and impulsivity as well as attention deficits, which reinforce chronic pruritus (CP) and skin picking disorder (SPD).

Taken together, these three features seem to be most prominent regarding the skin and may mutually reinforce each other (refer to Figure 2):

- Sensory processing issues: may result in (hyper)sensitive skin that is more prone to triggers related to CP.
- Hyperactivity and impulsivity: may result in SPD and certain secondary skin lesions like prurigo nodularis.
- Attention deficits: may worsen adherence problems regarding dermatological as well as psychotherapeutic interventions.

### 5.2. Neuroinflammatory Hypothesis

As already suggested for AD, neuroinflammatory cytokines, which mediate inflammation within the nervous system, can cross the blood–brain barrier and lead to changes in the maturation of structural and functional brain structures and neurotransmitter systems, possibly involved in ADHD pathology. For instance, altered levels of certain markers like interleukin (IL)-6, IL-4, IL-17A, and nerve growth factor have been identified as independent risk factors for ADHD in AD [2,41]. Additionally, neuroinflammation in ADHD has been linked to alterations in neurotransmitter systems, including the dopaminergic, serotonergic, and glutamatergic systems, which are sensitive to inflammatory processes [58]. Similarly, AD is characterized by chronic inflammation that can lead to systemic immune activation, potentially influencing neurodevelopmental outcomes [39].

The hypothalamus–pituitary–adrenal axis dysregulation is another common feature. Both AD and ADHD have been associated with altered cortisol responses, indicating a potential role of stress and immune system interactions in the comorbidity of these conditions [59].

Studies have also hypothesized genetic factors; however, in a recent Mendelian randomization analysis, no causal genetic relationship between AD and ADHD was found [60]. The authors of this study hypothesized that previously reported associations between AD and ADHD might have been due to confounding lifestyle factors including psychosocial stress and sleep disturbances. However, there might be similar epigenetic factors influencing neuroinflammation in AD and ADHD, including alterations in DNA methylation, histone modifications, and non-coding RNA regulation, which collectively influence inflammatory pathways and immune dysregulation [61,62,63,64,65,66].

Our two proposed hypotheses aim to shed light on possible pathophysiological concepts underlying the complex relationship between CP, ADHD, and SPD. However, as our observations stem from a non-experimental clinical routine, we cannot draw conclusions about potential causal relationships. This work explores potential links between ADHD, CP, and SPD in the described patient subgroup. While we propose a bidirectional, triangular relationship as a framework, these findings remain correlational and should be interpreted with caution. For instance, ADHD may act as an independent factor or mediator amplifying the itch–scratch cycle through impaired self-regulation and increasing impulsivity.

Concrete suggestions on how to manage patients presenting with a combination of these conditions might be of importance to clinicians. In the following, we therefore provide specific recommendations derived from our clinical experience with this patient group.

### 5.3. Management of CP, ADHD, and SPD in the Clinical Practice: Challenges and Opportunities

- *Recognize clinical signs:* ADHD often goes unrecognized, especially in adult women. In clinical practice, clinicians should look for signs like restlessness, rapid speech, frequent interruptions, and difficulty sustaining attention during consultation. Women with ADHD may also experience anxiety, low self-esteem [67,68], reduced social–emotional well-being, difficulty in relationships, and feelings of a lack of control [69]. Regarding SPD, patients may report an irresistible urge to scratch or manipulate their skin, even in the absence of itch—a key symptom of SPD.
- *Apply screening tools:* Most dermatologists lack direct access to mental health professionals. Simple screening tools can help identify comorbidities. Due to the high prevalence of depression and anxiety in CP, screening tools for depression—like the PHQ-8/9 or the Hospital Anxiety and Depression Scale (HADS) [70]—or anxiety—like the GAD-7 or HADS—have been recommended as screening tools [22,71]. As outlined in our work, ADHD and SPD can also be relevant comorbidities of CP that can be screened using the instruments provided in Table 3. However, clinicians should be aware that patients with severe CP may show agitation, overwhelm, and inattentiveness due to pruritus, which could be misinterpreted as ADHD symptoms. After a positive screening, ADHD or SPD should always be confirmed by a respective specialist.
- *Individualize treatment plans and medication:* While specific treatment guidelines exist for managing CP, ADHD, and bodily focused repetitive disorders like SPD (refer to Table 1 for references), managing all three conditions together remains challenging. Our three patients required individualized approaches based on unique dynamics and life circumstances. Patients’ adherence but also medication effectiveness varied greatly. ADHD was treated with methylphenidate in patients 1 and 3 (combined with escitalopram in patient 1). While in patient 1 symptoms of ADHD, SPD, and CP subsequently improved, pre-existing long-term treatment with methylphenidate did not prevent CP and SPD in patient 3. Patient 2 refused methylphenidate but showed improvement with N-acetylcysteine (NAC), reducing skin picking and CP, which is in accordance with the previous literature reporting positive effects of NAC on SPD [35,55,72], thereby indirectly benefitting CP through reduced skin damage [73]. Our observations on the inconsistent effects of methylphenidate in patients 1 and 3 align with those of the current literature. So far, two publications found reduced skin picking behavior after treating a 26-year-old woman and a 10-year-old boy, respectively, with methylphenidate [74,75]. This effect was explained by methylphenidate influencing the dopaminergic activity in the prefrontal cortex and striatum regions regulating impulse control and attention. According to another single case report, however, methylphenidate triggered skin picking in a 7-year-old boy with ADHD. Possible explanations for this contradictory finding are not discussed by the authors, however. Due to this scarce information from the medical literature, drawing general conclusions about the effectiveness of methylphenidate on SPD is very limited. Future studies exploring the effect of methylphenidate on SPD in a larger sample are needed.
- *Combine with psychotherapy*: Very often, medication alone is insufficient in treating patients with ADHD and/or SPD. Additional psychotherapy helps manage condition-specific symptoms and develop functional coping strategies. Our patients received pruritus-specific, in-house CBT, which has been shown to be effective for CP patients [76]. Two of our patients began outpatient ADHD- and SPD-focused CBT. Psychodynamic therapy may also be helpful in exploring possible underlying functions of maladaptive behavioral patterns. In addition, it should also be mentioned that ADHD and trauma-related disorders show clinical overlap [77,78]. Therefore, bidirectional links between CP, SPD, and other mental disorders are at least conceivable.
- *Address treatment adherence:* ADHD-related inattention and disorganization can hinder adherence to treatment regimens, including topical or systemic medications. Offering a simple regimen, being patient, and maintaining an open and trusting patient–doctor relationship can improve treatment adherence.
- *Provide regular follow-up bi-disciplinary consultations:* Due to fluctuating severity, influenced by physiological as well as internal and external psychosocial factors, our patients continued regular bi-disciplinary follow-up consultations to discuss treatment options and address or react to new somatic or psychosocial aspects. A collaborative approach of dermatologists and mental health specialists is vital to meet the complex needs of this patient group [79,80]. Our bi-disciplinary setting made our patients feel understood and cared for. Patient 1, for example, found strong relief after receiving an ADHD diagnosis and professional help.

## 6. Conclusions

Based on our three cases and the discussion above, we believe that there is an intricate interaction between CP, ADHD, and SPD in a subgroup of patients. Each of these conditions stands in a bidirectional relationship with the other two. This concept is supported by the medical literature and may be grounded in neurodevelopmental and neuroinflammatory commonalities. Neurodevelopmental alterations can disrupt sensory processing and executive functions, with (neuro)inflammatory processes possibly playing a central role. Key features include (1) sensory processing problems leading to (hyper)sensitive skin that is prone to CP, (2) hyperactivity and impulsivity manifesting as skin picking, and (3) attention deficits and other executive dysfunctions worsening treatment adherence. Bi-disciplinary collaboration between dermatologists and mental health specialists is therefore crucial for addressing these complex patients.

## Figures and Tables

**Table 2:** Overview of the three cases regarding patient characteristics, diagnoses, individual scores from applied quantitative and qualitative itch-related assessments and mental health-related screenings (German versions), and newly initiated treatment.

	Range	Case 1	Case 2	Case 3
Sex		Female	Female	Female
Age (years)		53	69	51
**Diagnoses**				
Chronic Pruritus (CP)	-	✓ (IFSI III)	✓ (IFSI III)	✓ (IFSI II)
Attention-Deficit/Hyperactivity Disorder (ADHD)	-	✓	✓	✓
Skin Picking Disorder (SPD)	-	✓	✓	✓
**Pruritus-specific Assessments**				
Duration of CP (Years)	-	6	10	2
Mean Itch Intensity (Past 4 Weeks): Numeric Rating Scale (NRS)	0–10	7	5	8
Itch-related Quality of Life: GerItchyQoL	0–110	75	57	85
Laboratory Pruritus Screen (According to [10])	-	Total IgE: 340 kIU/L, otherwise unremarkable	Total IgE: 490 kIU/L, Inhalation screen: sx1: 2.63 kIU/L	TSH: 5.57 mIU/L
**Mental Health-specific Assessments**				
ADHD:				Not applied due to already confirmed diagnosis.
ADHS-SB	0–66	25	35	
Skin Picking:				
mSPS-D	0–36	26	20	18
Depression:				
PHQ-8	0–24	7	0	15
Anxiety:				
GAD-7	0–21	4	5	9
Somatic Burden:				
SSS-8	0–32	7	1	23
SSD-12	0–48	22	16	40
**Newly Initiated Treatment**				
Dermatological	-	Topical: mometasone (0.1%), pimecrolimus, polidocanol Systemic: hydroxyzine (25 mg/d, in the evening)	Topical: polidocanol and clobetasol (0.5 mg/g) Systemic: *N*-acetylcysteine (600 mg, twice/d)	Topical: mometasone (0.1%), polidocanol Systemic: bilastine (20 mg) Device-based: NB-UVB light therapy
Mental	-	Pharmacological: methylphenidate (5 mg/d), escitalopram (10 mg/d) Psychotherapy: in-house and external CBT	Pharmacological: none Psychotherapy: in-house CBT	Pharmacological: methylphenidate (10 mg/d) Psychotherapy: in-house and external CBT

Abbr.: ADHS-SB: ADHD self-report questionnaire, taken from the German Homburger ADHD Scale for Adults (HASE) [28]; CBT: cognitive behavioral therapy; IFSI: International Forum for the Study of Itch [4]. GerItchyQoL: German version of the ItchyQoL [29]; mSPS-D: modified version of the Skin Picking Scale [30]; PHQ-8: Patient Health Questionnaire with 8 items [31]; GAD-7: General Anxiety Disorder questionnaire [32]; SSS-8: Somatic Severity Scale [33]; SSD-12: Somatic Symptom Disorder Criteria B-scale [34].

**Table 3:** Possible screening instruments for attention-deficit/hyperactivity disorder (ADHD) and skin picking disorder (SPD).

	ADHD	SPD	
**Instrument**	**Adult ADHD Self-Report Scale (ASRS-5)** [81]	**Skin Picking Scale-Revised (SPS-R)** [82]	**Skin Picking Impact Scale (SPIS)** [83]
**German version**	German version by Ballmann and colleagues [84].	Modified German version (mSPS-D) by Mehrmann and colleagues [30].	German version (SPIS-D) by Mehrmann and colleagues [30].
**Description**	Assesses with DSM-5 criteria for ADHD. Can be used in primary care and other non-specialist settings.	Assesses the frequency, intensity, and burden of SPD.	Assesses psychosocial impairment caused by skin picking.
**Number of items, range**	German ASRS-5: 6, 0–30	mSPS-D: 9, 0–36	SPIS-D: 4, 0–16
**Clinical cut-off**	Positive screening for ADHD with a clinical cut-off of ≥14.	For the original version: clinical cut-off of 9 [85]. So far, no official cut-off scores exist for the German version.	For the original version: clinical cut-off of 7 [83]. So far, no official cut-off scores exist for the German version.
